# Glomerular and Tubular Functions in Children and Adults with Transfusion-Dependent Thalassemia

**DOI:** 10.4274/tjh.2017.0266

**Published:** 2018-03-06

**Authors:** Agageldi Annayev, Zeynep Karakaş, Serap Karaman, Altan Yalçıner, Alev Yılmaz, Sevinç Emre

**Affiliations:** 1 İstanbul University İstanbul Faculty of Medicine, Department of Pediatric Hematology and Oncology, İstanbul, Turkey; 2 Düzen Laboratories, İstanbul, Turkey; 3 İstanbul University İstanbul Faculty of Medicine, Department of Pediatric Nephrology, İstanbul, Turkey

**Keywords:** Thalassemia, Tubulopathy, Glomerulopathy, β2-Microglobulin, Cystatin

## Abstract

This study aimed at assessing renal functions in patients with transfusion-dependent thalassemia (TDT). Fifty patients and 30 controls were enrolled in this prospective study. Serum levels of electrolytes and albumin were measured by a spectrophotometer. Serum levels of cystatin-C and urinary levels of β2-microglobulin were measured by nephelometric method. Thirty-eight patients were receiving deferasirox and 8 were on deferiprone. Serum electrolytes and albumin levels of the patients were found to be within normal ranges. Urinary β2-microglobulin and serum cystatin-C levels were significantly higher in patients than controls. They did not significantly differ between the subgroup of patients on deferiprone and the control group, whereas they were found to be higher in patients using deferasirox compared to controls. Urinary β2-microglobulin levels significantly increased in patients who were receiving high-dose deferasirox compared to those who were receiving a daily dose of 15-20 mg/kg or controls. Subclinical renal injury may be present in TDT patients.

## Abstract (tr)

Bu çalışmada transfüzyona bağımlı talasemi (TBT) hastalarında böbrek fonksiyonlarının değerlendirilmesi amaçlanmıştır. Prospektif çalışmaya, 50 TBT ve 30 kontrol grubu dahil edildi. Serum elektrolitleri ve albumin düzeyleri spektrofotometre ile ölçüldü. Serum sistatin-C ve idrar β2-mikroglobülin düzeyleri nefelometrik yöntemle ölçüldü. Otuz sekiz hasta deferasiroks, 8 hasta deferipron alıyordu. Hastaların serum elektrolitleri ve albumin düzeyleri normal sınırlardaydı. İdrar β2-mikroglobulin ve serum sistatin-C düzeyleri hasta grubunda kontrol grubundakilere göre anlamlı derecede yüksekti. Serum Cys-C ve idrar β2-mikroglobulin düzeyleri, deferipron kullananlar ve kontrol grubu arasında anlamlı farklılık göstermezken, deferasiroks kullananlarda kontrol grubuna göre daha yüksek bulundu. İdrar β2 mikroglobulin düzeyleri, yüksek doz deferasiroks alan hastalarda, 15-20 mg/kg/gün deferasiroks alanlara veya kontrol grubuna göre anlamlı şekilde artmıştı. Transfüzyona bağımlı talasemi hastalarında subklinik olarak renal hasar mevcut olabilir.

## Introduction

Iron accumulation may lead to renal damage in transfusion-dependent thalassemia (TDT) [1,2]. Cystatin C (Cys-C), a small molecular weight protein, is filtered from the glomeruli, reabsorbed from the tubular cells, and metabolized from the kidneys. It is a good marker for glomerular filtration rate (GFR). b2-Microglobulin (b2MG), a single-chain, low-molecular-weight polypeptide, is filtered by the glomeruli, then reabsorbed and catabolized in the proximal tubular cells. Increased urinary excretion of b2MG may demonstrate tubular dysfunction. Our study assessed kidney function in patients with TDT using serum Cys-C (SCys-C) and urinary b2MG (Ub2MG) measurements in addition to routine tests, as well as the utility of these markers as indicators for early glomerulopathy and tubulopathy.

## Materials and Methods

Fifty patients with TDT, 25 under and 25 above the age of 18, have been followed since their childhood by the Thalassemia Center at the İstanbul Faculty of Medicine of İstanbul University. Twenty-two patients were male and 28 were female. The mean age was 18.4±11.8 years (range: 2-45). Thirty age- and sex-matched subjects were included in the control group. Ethical approval was granted by the institutional review board and patient consent was obtained. 

Serum electrolytes, urine calcium/creatinine (uCa/Cr) and fractional excretion of sodium (FENa), GFR, proteinuria, serum Cr, and albumin levels were measured and were compared to their reference ranges in the patient group. SCys-C and Ub2MG levels in the patient group were compared to those of the controls and potential correlations between SCys-C or Ub2MG levels and the severity of anemia, ferritin levels, and chelation therapy were also evaluated. Blood samples collected to measure SCys-C were cold-centrifuged at 4000 rpm for 10 min. A photometric analysis of serum albumin, urea, Cr, uric acid, and electrolyte levels was conducted. Ferritin levels were measured by the electrochemiluminescent immunoassay method. SCys-C and Ub2MG levels were measured by nephelometry. 

Statistical analysis was done using SPSS 17.0.

## Results

No significant differences were found between the patients and controls in terms of age or sex (p>0.05). Demographic and clinical characteristics of the patients are summarized in Table 1. In the patients, serum Na, K, Ca, P, Mg, urea, Cr, and albumin were within normal limits. None of the patients had proteinuria. The mean uCa/Cr ratio was found to be higher than the normal level. Estimated GFR was elevated in the patient group (Table 2). SCys-C and Ub2MG levels were higher in patients than controls (p=0.001, p=0.010) (Table 3). SCys-C was increased with age (r=0.376; p=0.007). No correlations were found between Ub2MG levels and age (r=-0.186, p=0.217). 

**Figure 1:**
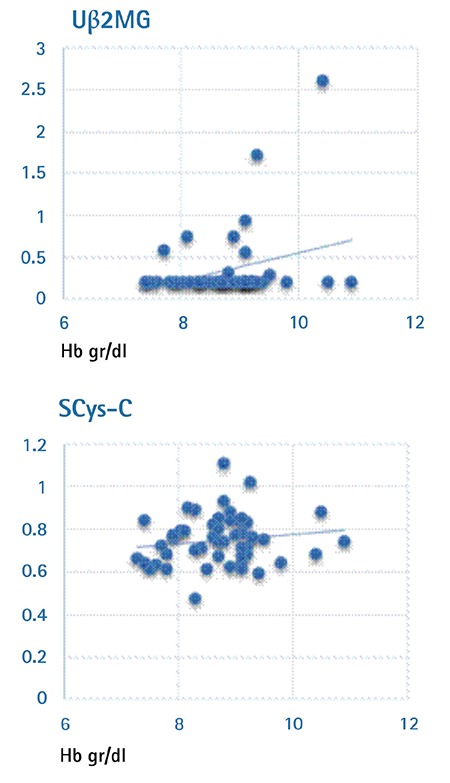
Correlations between pretransfusion hemoglobin and urinary β2-microglobulin and serum cystatin-C. *Uβ2MG: Urinary β2-microglobulin, SCys-C: serum cystatin-C, Hb: hemoglobin.*

**Figure 2:**
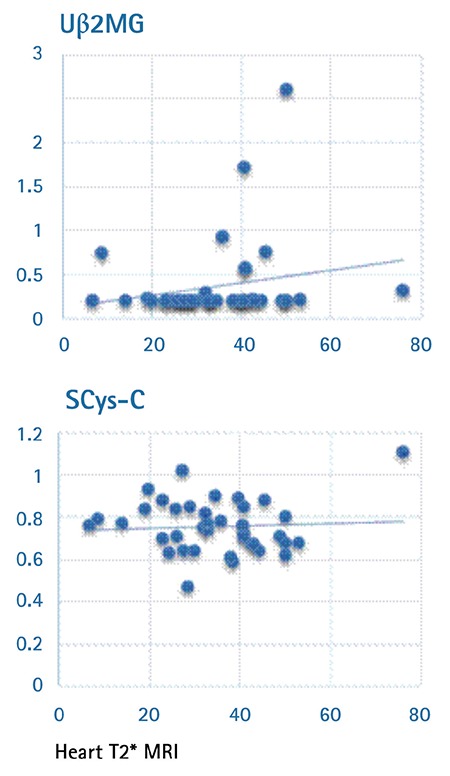
Correlations between heart T2* and Uβ2MG and SCys-C. *Uβ2MG: Urinary β2-microglobulin, SCys-C: serum cystatin-C, MRI: magnetic resonance imaging.*

Renal dysfunction was detected in 30 out of 50 patients. FENa levels were increased in 8 patients, while Ub2MG and SCys-C levels were increased in 9 and 25 patients, respectively. In our study, renal involvement was observed in 54% of the patients under the age of 18 and 64% of the patients above the age of 18. No correlations were observed between the mean SCys-C and Ub2MG levels and pretransfusion hemoglobin and iron load (p>0.05) (Figures 1 and 2). No correlations were found between SCys-C and ferritin levels. The assessment of the correlations between the Ub2MG and ferritin levels in patients revealed that Ub2MG levels were greater than in the controls, particularly in those who had a ferritin level of <500 ng/mL or >1000 ng/mL (p=0.001). Although Ub2MG levels were slightly elevated in the patients who had ferritin levels between 500 and 1000 ng/mL, the difference between the patients and controls was not statistically significant (p>0.05). 

The distribution of patients by their chelation therapy was as follows: 38 patients (75.5%) were on deferasirox (DFX); 8 (16.3%) were on deferiprone (DFP). Among the patients who were on DFX, 11 (31%) were receiving a dose of 15-20 mg/kg/day, 13 (33%) were receiving a dose of 20-30 mg/kg/day, and 14 (36%) were receiving a dose of 30-40 mg/kg/day. When SCys-C concentrations were categorized by iron chelation therapy, there were no differences between the patients who were on DFP and the controls, while significant differences were found between the patients who were on DFX and the controls (p=0.002) (Table 4). No correlations were found between DFX dosages and SCys-C concentrations. When urinary b2MG levels were categorized by iron chelation therapy, there were no differences between the patients who were on DFP and the controls, while significant differences were found between the patients who were on DFX and the controls (p=0.004) (Table 4). In the subgroup of patients on DFX, the assessment of Ub2MG and SCys-C levels by DFX doses revealed that there were no significant differences between the controls and patients who were taking DFX at a dose of 15-20 mg/kg, while statistically significant differences were found between controls and patients who were taking DFX at a dose of 20-40 mg/kg (p=0.011). Ub2MG levels were increased with increasing DFX doses. SCys-C levels were higher in all patient groups in comparison to the control group (p=0.013), but the difference was not dose-related.

## Discussion

In our study, serum electrolytes were within reference ranges, but FENa levels were elevated in 8 patients. In another study, FENa was elevated in 29% of 103 TBT patients [3]. Several studies have reported normal FENa levels [4,5]. In our study the Ca/Cr ratio was found to be higher than the upper limit of the normal range in 28% of the patients. Higher Ca/Cr ratios may be associated with tubular dysfunction as well as with impaired calcium homeostasis or bone disorders. In our study, osteoporosis was diagnosed in almost half of the patients. Some studies have reported high levels of Ub2MG in patients with TBT [6,7,8], while other studies reported the opposite [5]. In our study, Uβ2MG concentrations in patients were significantly higher than in the controls. No significant differences were found between the controls and the subgroup of patients who were on DFP, whereas statistically significant differences were found between the controls and the DFX subgroup. Positive correlations between the Uβ2MG levels and DFX doses suggested that DFX might cause dose-dependent tubulopathy. Uβ2MG levels were significantly higher in patients than the controls, particularly in patients who had ferritin levels of <500 ng/mL or >1000 ng/mL, whereas even though Uβ2MG levels were slightly elevated in the subgroup of patients with ferritin between 500 and 1000 ng/mL, the difference between this subgroup and the control group was not statistically significant. No associations were found between Uβ2MG levels and iron load.

GFR has been a commonly used method to measure kidney functions. In two studies, no significant differences were found in GFR between patients and controls [3,9]. In our study, GFR in the patient group was higher than the upper limit of the age-adjusted reference range. The routine markers of kidney function, including serum urea and Cr levels, were within normal limits in all patients, in line with similar studies in the literature [10,11,12].

Some studies reported higher SCys-C levels in patients with TBT [13,14,15]. In our study, SCys-C levels were found to be significantly higher in the patients in comparison to the controls. No differences were found between the patients who were taking DFP and the controls, while SCys-C levels were significantly higher in patients on DFX in comparison to the controls. No correlations were found between SCys-C or ferritin levels and pretransfusion Hb, liver, and heart T2* values, while SCys-C levels increased with age. Koliakos et al. [16] revealed that the urinary markers of tubular dysfunctions correlated positively with serum ferritin and liver iron deposition in patients with TBT. Papassotiriou et al. [17] detected elevated SCys-C in patients who received DFX at doses of 20-40 mg/kg/day. Acute kidney injury has been reported in 40% of patients on deferoxamine [18]. None of our patients were taking deferoxamine during this study.

## Conclusion

In conclusion, patients with TDT may develop renal dysfunction. In follow-up, regular testing for early markers in addition to routine kidney function tests may be beneficial to prevent future severe kidney dysfunction.

## Figures and Tables

**Table 1:**
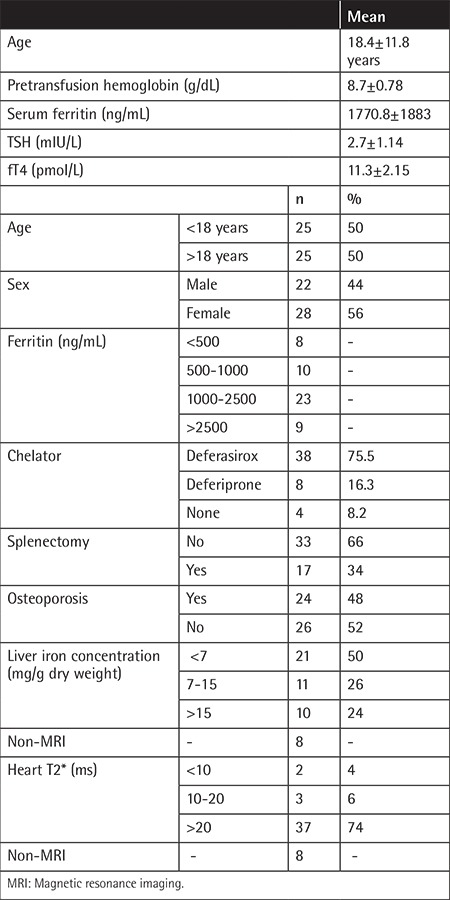
Demographics and clinical characteristics of the patient group.

**Table 2:**
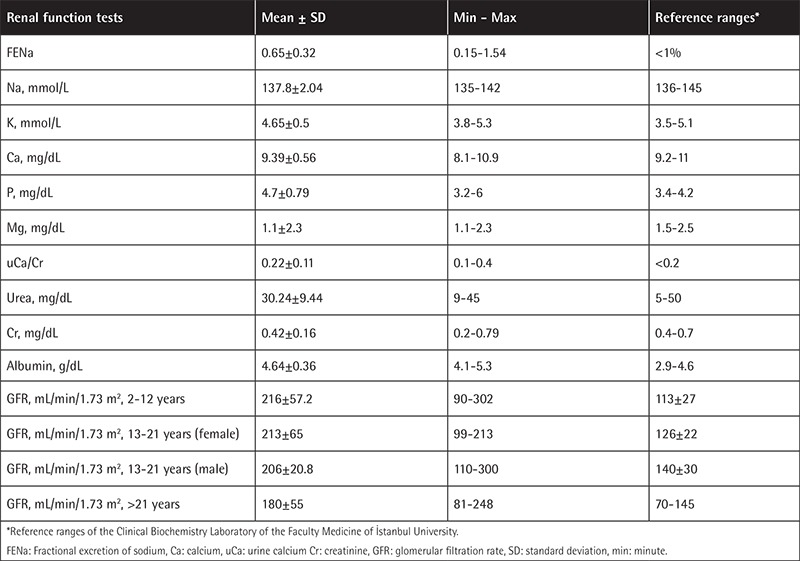
Renal function test results in the patient group and reference ranges.

**Table 3:**
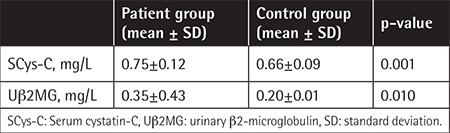
Serum cystatin-C and urinary β2-microglobulin levels in the patient and control groups.

**Table 4:**
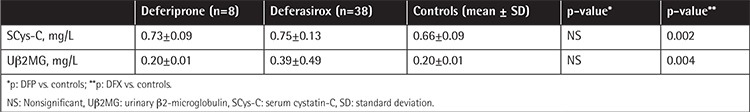
The comparisons of urinary β2-microglobulin and serum cystatin-C levels between the control group and the subgroups of patients who were on chelation therapy with deferiprone or deferasirox.
